# Oncologic Outcomes of Patients with Early-Stage Cervical Cancer after Minimally Invasive Radical Hysterectomy and Sentinel Lymph Node Biopsy

**DOI:** 10.3390/jcm13133981

**Published:** 2024-07-08

**Authors:** Tomohito Tanaka, Ruri Nishie, Hikaru Murakami, Hiromitsu Tsuchihashi, Akihiko Toji, Shoko Ueda, Natsuko Morita, Sousuke Hashida, Shinichi Terada, Hiroshi Maruoka, Kohei Taniguchi, Kazumasa Komura, Masahide Ohmichi

**Affiliations:** 1 Department of Obstetrics and Gynecology, Educational Foundation of Osaka Medical and Pharmaceutical University, 2-7 Daigakumachi, Takatsuki 569-8686, Osaka, Japan; ruri.nishie@ompu.ac.jp (R.N.); hikaru.murakami@ompu.ac.jp (H.M.); hiromitsu.tsuchihashi@ompu.ac.jp (H.T.); akihiko.touji@ompu.ac.jp (A.T.); shouko.ueda@ompu.ac.jp (S.U.); natsuko.morita@ompu.ac.jp (N.M.); sosuke.hashida@ompu.ac.jp (S.H.); shinichi.terada@ompu.ac.jp (S.T.); hiroshi.maruoka@ompu.ac.jp (H.M.); m-ohmichi@ompu.ac.jp (M.O.); 2 Center for Medical Research & Development, Division of Translational Research, Osaka Medical and Pharmaceutical University, 2-7 Daigakumachi, Takatsuki 569-8686, Osaka, Japan; kohei.taniguchi@ompu.ac.jp (K.T.); kazumasa.komura@ompu.ac.jp (K.K.)

**Keywords:** cervical cancer, laparoscopy, minimally invasive surgery, radical hysterectomy, sentinel node biopsy

## Abstract

**Background**: The sentinel lymph node is the first node that cancer cells reach when migrating from the primary site. However, oncological outcomes after sentinel lymph node biopsy (SNB) have not been reported for cervical cancer. In this study, oncological outcomes were compared between patients receiving SNB and pelvic lymphadenectomy (PLD) for early-stage cervical cancer. **Methods**: One hundred and four patients with clinical stage 1A2, 1B1, and 2A1 cervical cancer were included in this study. All patients underwent laparoscopic or robot-assisted radical hysterectomy with SNB or PLD. Fifty-two patients with tumors ≤2 cm underwent SNB. Disease-free survival (DFS) and overall survival (OS) were compared between the groups. **Results**: The median (interquartile range) tumor size was 12 (7–20) mm in the SNB group and 20 (13–25) mm in the PLD group. Lymph node metastasis occurred in one patient in the SNB group and in nine patients in the PLD group. The median follow-up periods were 42 (24–60) and 82 (19–101) months in the SNB group and PLD group, respectively. The 3-year DFS rates were 100% in SNB and 91.5% in PLD. The 3-year OS was 100% in both groups. **Conclusions**: SNB was sufficient in cervical cancer patients with tumors ≤2 cm, suggesting that PLD might not be necessary for these patients.

## 1. Introduction

Abdominal radical hysterectomy with pelvic lymphadenectomy (PLD) is the standard surgical treatment for patients with cervical cancer [1]. However, the rate of lymph node metastasis is low in small-size cervical cancers, and PLD may result in overtreating these patients. The sentinel lymph node (SLN) is the primary node that cancer cells reach when they metastasize. Therefore, SLN has the highest rate of metastasis among regional lymph nodes. Consequently, the rate of lymph node metastasis is presumably low when no metastases are observed in SLN. Sentinel lymph node biopsies (SNBs) are performed worldwide as part of the treatment of cervical cancer, while detection rate and sensitivity are estimated at 89.2% and 90%, respectively. However, the oncological outcomes have not been reported [2]. In this study, these outcomes were compared between patients receiving SNB and PLD for early-stage cervical cancer.

## 2. Materials and Methods

### 2.1. Participants

This was a prospective cohort study of patients with early-stage cervical cancer who underwent minimally invasive surgery with either SNB or PLD. All patients underwent robotic or laparoscopic radical hysterectomy, with or without bilateral salpingo-oophorectomy, at the Educational Foundation of Osaka Medical and Pharmaceutical University, Japan, from January 2013 to December 2023. Patients who underwent PLD with SNB between 2013 and 2016 were included. After 2017, patients with a tumor size of ≤2 cm underwent sentinel node navigation surgery (SNNS), while those with tumors >2 cm underwent PLD. PLD was performed when metastasis was found in the SLN upon frozen section diagnosis during surgery. When revealed after surgery, the patient receives radiotherapy. Laparoscopic and robotic radical hysterectomies were performed via vaginal closure. This study was approved by the Osaka Medical College Clinical Research Review Board (2013-053, 2015-111, 2018-082, 2020-087), and written informed consent was obtained from all participants.

Patients who met the following criteria were eligible for inclusion in this study: (1) cervical cancer International Federation of Gynecology and Obstetrics (FIGO) (2009) stage 1A2, 1B1, or 2A1; (2) patients who did not present any lymph node metastasis on preoperative imaging; (3) laparoscopic or robotic radical hysterectomy (type 3 with nerve sparing) with SNB or PLD for cervical cancer; and (4) patients without previous chemotherapy or radiotherapy.

### 2.2. Diagnosis and Adjuvant Therapy

All patients were diagnosed according to FIGO (2009). After hysterectomies, pathological evaluations including histological type, tumor size, and several risk factors for recurrence were performed. Patients with risk factors for recurrence, including large tumor size (>4 cm), lymph node metastasis, parametrial invasion, deep stromal invasion (more than half), lymphovascular space invasion, and a positive-cut vaginal end, received adjuvant therapy.

### 2.3. SNB Procedure

On the day before surgery, 2.0 mL of fluid containing 110 MBq 99m-Technetium (^99m^Tc)-labeled tin colloids were injected into the patient’s cervix. On the day of surgery, 5 mL of indigocarmine (IDC) (4 mg/mL) and indocyanine green (ICG) (50 µg/mL) were also injected into the cervix at the start of the operation. After entering the retroperitoneal cavity, SLNs were detected by direct inspection or using a gamma probe or a color fluorescence camera. In the SNB group, PLD was omitted when the frozen section of the SLN was negative. The cardinal lymph nodes were dissected from all patients. After 2017, we performed SNB with the removal of the lymphatic vessel. The lymphatic vessels between the uterus and SLN were completely removed using SLN [3].

### 2.4. Radical Hysterectomy Procedure

Most patients underwent laparoscopic radical hysterectomy (type 3 nerve-sparing) with the vaginal closure method without intrauterine manipulation. Six patients underwent robot-assisted radical hysterectomy. The intra-abdominal procedures for laparoscopic and robotic radical hysterectomy were identical. The uterus was retracted using 5 mm grasping forceps, which were inserted into the left side under the costal arch. After SNB or PLD, a radical hysterectomy was performed, with or without salpingo-oophorectomy. The round and broad ligaments were then coagulated and transected. After clamping the uterine artery lateral to the ureters and opening the ureteral tunnels, the ureters were unroofed and rolled laterally. The infundibulopelvic ligaments were coagulated and transected bilaterally. The vesicouterine and uterosacral ligaments were resected. After the dissection of the cardinal nodes, the deep uterine vein was isolated and clamped. The rectovaginal septum was then separated. After the inferior hypogastric nerve was identified and divided laterally, the bilateral paracolpium was performed using a 1-0 polydioxanone suture. The corresponding paracolpos was resected. Transvaginal vaginal closure was performed immediately before the vagina was entered. The vagina was then entered on a laparoscope, the remaining vaginal tissue was cut, and a circumferential colpotomy was performed. After removing the uterus with or without the adnexa, the vaginal cuff was closed laparoscopically using running absorbable sutures.

### 2.5. Evaluation of Lymph Nodes

The removed SLN were cut half-parallel to the longest axis to evaluate the maximum sectional area. Half of the tissue was used for frozen section diagnosis. The remaining half of the tissue was cut every 2 mm into 5 µm thick sections and then stained with hematoxylin and eosin (H&E). The removed lymph nodes, except SLN, were cut in half parallel to the longest axis and stained with H&E.

### 2.6. Statistical Analysis

JMP software (version 15.1.1) (SAS Institute Japan, Tokyo, Japan) was used for all statistical analyses. Continuous variables were expressed as median with interquartile range and compared using the Mann–Whitney U-test. Fisher’s exact test was used to compare frequencies. Survival was estimated using the Kaplan–Meier method with a log-rank test. Statistical significance was set at *p* < 0.05.

## 3. Results

**Figure 1:**
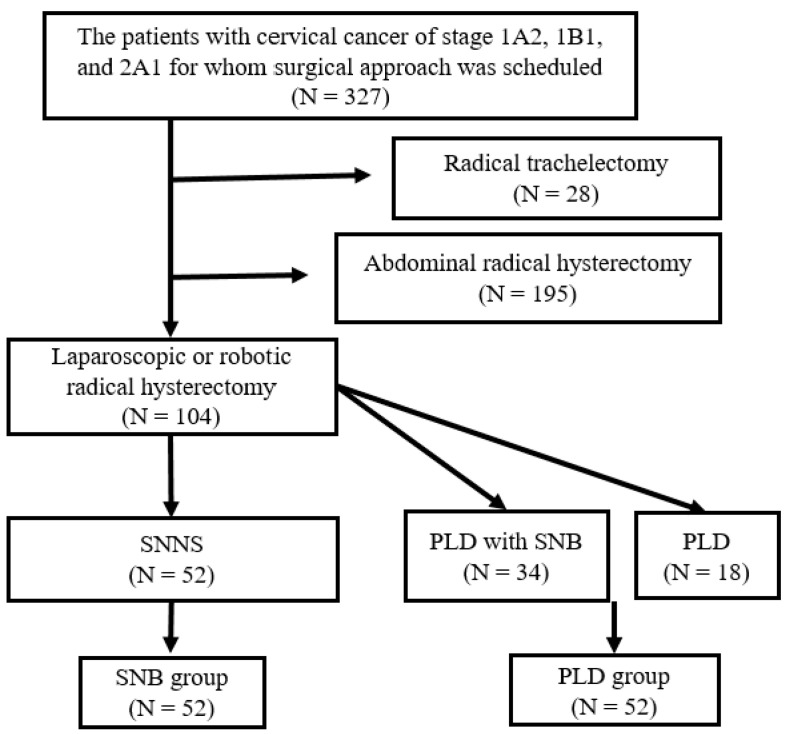
Chart of the study participants. Among 327 patients with cervical cancer of stage 1A2, 1B1, and 2A1 and scheduled surgical approach, 28 underwent radical trachelectomy and 195 abdominal radical hysterectomy. The remaining 104 patients underwent laparoscopic or robotic radical hysterectomy. Sentinel node navigation surgery (SNNS) and pelvic lymphadenectomy (PLD) after sentinel lymph node biopsy (SNB) were performed in 52 and 34 patients, respectively. Eighteen patients underwent PLD only.

Figure 1 shows a chart of the study participants. Among 327 patients with stage 1A2, 1B1, and 2A1 cervical cancer who were scheduled for surgery, 104 met the inclusion criteria. There were 52 patients in each of the two groups.

Table 1 shows the characteristics of the study participants. In total, 104 patients were included in this study. Among them, 52 patients underwent SNB and 52 PLD with or without SNB (included in the PLD group). The rate of SLN detection in the SNB group was 100%. The median age was 46 (41–70) years in the SNB group and 45 (36–51) years in the PLD group (*p* = 0.3). In the SNB group, eight patients had cervical cancer stage 1A2, forty-three had stage 1B1, and one had stage 2A1. In the PLD group, three patients had stage 1A2 cervical cancer, forty-three had stage 1B1, and six had stage 2A1. Overall, the PLD group had a higher tumor stage than the SNB group (*p* = 0.03). Histologically, 24 and 27 squamous cell carcinomas and 28 and 25 adenocarcinomas were detected in the SNB and PLD groups, respectively. The median tumor size was 12 (7–20) mm in the SNB group and 20 (13–25) mm in the PLD group. Overall, the SNB group had smaller tumors than the PLD group (*p* = 0.001). None of the patients in the SNB group had large tumors (>4 cm), and only one patient had a large tumor in the PLD group. There were one and nine lymph node metastases in the SNB and PLD groups, respectively. Among a total of ten patients who had lymph node metastasis, six underwent SNB, while lymph node involvement in the SLN was observed in all six patients. None of the patients in the SNB group showed parametrial invasion, whereas there were two patients in the PLD group in whom invasion was detected. There were seven and seventeen patients with deep stromal invasion, while five and thirteen patients presented lymphovascular space invasion in the SNB and PLD groups, respectively. None of the patients in the SNB group had a positive vaginal cut, while this was present in one patient in the PLD group. Seven and twenty-eight patients received adjuvant therapy in the SNB and PLD groups, respectively. The median follow-up period was 42 (24–60) months in the SNB group and 82 (19–101) months in the PLD group. The minimum follow-up period was six months in both groups. The proportions of patients with less than 24 months of follow-up were 27% and 31% in SNB and PLD groups, respectively.

**Figure 2:**
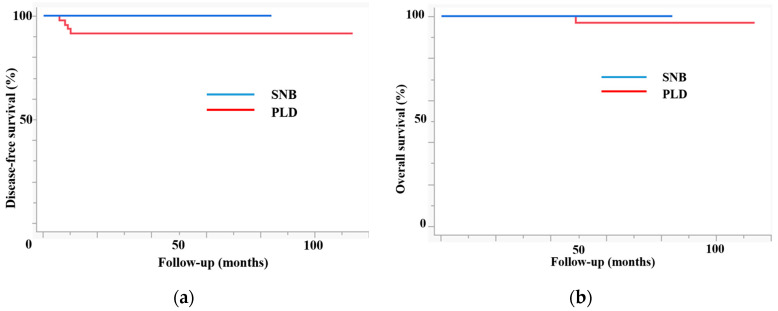
The prognosis of patients in both groups. (**a**) Disease-free survival. (**b**) Overall survival. The sentinel lymph node biopsy (SNB) group had higher disease-free survival (3 y DFS, 100% vs. 90.6%, *p* = 0.04) and overall survival (3 y OS, 100% vs. 100%, *p* = 0.05).

Figure 2 shows the prognosis of patients in both groups. The SNB group had higher disease-free survival (DFS) (3-year DFS, 100% vs. 90.6%, *p* = 0.04) and overall survival (OS) rates (3-year OS, 100% vs. 100%, *p* = 0.05). As expected, patients in the SNB group had a better prognosis than those in the PLD group because the former included patients with more advanced disease. In this study, there was no disease recurrence, while the mortality rate was zero in the SNB group.

Table 2 shows the clinical characteristics of the four patients with disease recurrence. All four patients had stage 1B1 disease. There were two squamous cell carcinomas and two adenocarcinomas. The tumor sizes ranged from 11 to 20 mm. In case 1, the patient had deep stromal and lymphovascular space invasion. The remaining patients had no pathological risk factors for recurrence. No lymph node metastasis was observed in any of the patients with disease recurrence. In case 1, the patient received CCRT as adjuvant therapy. The other patients did not receive any adjuvant therapy. The duration between surgery and disease recurrence ranged from six to ten months. The sites of recurrence were the lungs, pelvis, and vagina. One patient died of the disease, while three were alive.

Table 3 reveals the subgroup analysis of the patients who had a tumor size of ≤2 cm. The SNB and PLD groups included 44 and 30 patients, respectively. The median tumor sizes were 10 (7–17) mm and 15 (10–18) mm in the SNB and PLD groups, respectively. Most patients in this subgroup had stage 1B1 disease. The patients in the PLD group presented more pathological risk factors than those in the SNB group. The rate of adjuvant therapy was 6.8% in the SNB group and 33.3% in the PLD group. No recurrences were observed in the SNB group. In contrast, there were four patients with disease recurrence in the PLD group. The 3-year DFS was 100% and 86.4% for SNB and PLD groups, respectively, while the 3-year OS was 100% in both groups.

**Figure 3:**
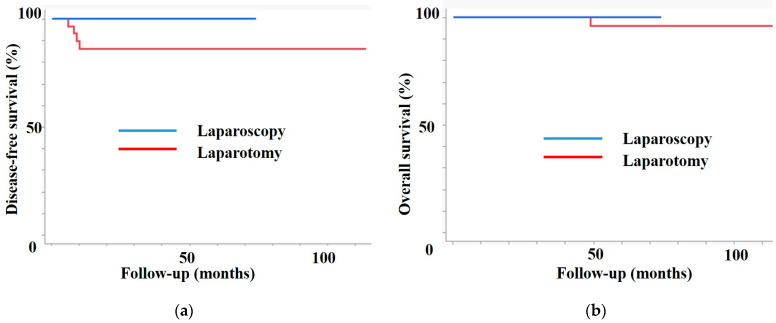
Subgroup analysis. (**a**) Disease-free survival. (**b**) Overall survival. In the subgroup of patients with a tumor of ≤2 cm, the sentinel lymph node biopsy (SNB) group had a better prognosis than the pelvic lymphadenectomy (PLD) group.

Figure 3 shows the prognosis of both groups in the subgroup analysis. Patients in the SNB group had higher DFS (3-year DFS, 100% vs. 86.4%, *p* = 0.02) and OS (3-year OS, 100% vs. 100%, *p* = 0.4) than patients in the PLD group.

## 4. Discussion

With a median follow-up of 42 months, no recurrence or death occurred in the SNB group. Based on the results of the current study, we concluded that SNB could be sufficient for patients with cervical cancer with tumors ≤2 cm, while PLD could be omitted.

A meta-analysis showed that the pooled detection rate and sensitivity of an SLN were 89.2% and 90%, respectively. Moreover, the detection rate and sensitivity were 93.4% and 94.7% in tumors ≤2 cm and 73.9% and 81.7% in tumors > 2 cm, respectively [4]. The detection rates of an SLN in the unilateral pelvis were 95.7% and 100%, while in the bilateral pelvis, these rates were estimated at 80.4% and 90% for technetium-99m (Tc) with and without blue dye and indocyanine green (ICG), respectively [5]. The pooled specific side detection rates were 85% in tumors ≤2 cm, 67% in tumors >2 cm, 75.2% for blue dye, 74.7% for Tc, 99.8% for the combined technique, and 85.5% for ICG [2]. A tumor diameter of ≤2 cm was considered a suitable indicator.

In a meta-analysis, the incidence of lymphedema was compared in 323 and 277 patients who underwent SNB and PLD, respectively. This incidence was significantly lower in the SNB group than in the PLD group (odds ratio (OR): 0.12 95% confidence interval (CI): 0.03 –-0.49) [5]. In a national prospective multicenter study, 200 of 245 patients with cervical cancer completed questionnaires preoperatively and at 3, 12, and 36 months postoperatively. The incidence of lymphedema was significantly lower in the SNB than in the SNB with PLD groups (5.6% vs. 32.3%) [6].

SNB confers with feasible accuracy and fewer complications compared with the systematic PLD described above. An important question is whether systematic PLD offers an additional therapeutic or survival advantage. A meta-analysis including a total of 1952 patients (383 underwent SNB and 1569 underwent PLD) showed that there was no significant difference in DFS (OR 1.04, 95% CI 0.66–1.66) or in OS (OR 0.99, 95% CI 0.46–2.45) between SNB and PLD [7]. The SENTICOL-2 trial is the largest randomized controlled trial to compare the effects of SNB and PLD. In total, 206 patients with early cervical cancer were included in that study. The 4-year DFS was estimated at 89.5% and 93.1% (*p* = 0.53) for SNB and PLD, respectively. Additionally, the 4-year OS rates were 95.2% and 93.1% (*p* = 0.97) for SNB and PLD, respectively [8]. However, the recurrence rate was higher in the SNB group (10.5% vs. 6.9%, *p* = 0.37). In prospective and retrospective observational studies on SNB, of 277 patients who underwent SNB, no recurrence was observed, with a median follow-up of 40–53 months [9,10,11]. Furthermore, in a retrospective study that included 110 and 1078 patients who underwent SNB and PLD, respectively, the 5-year recurrence-free survival was 93% for SNB and 92% for PLD. In a recently published meta-analysis, 5-year DFS and OS after SNB was more than 90%, while this rate was similar after PLD. Ultra-staging did not affect DFS [12]. These data suggest that SNB may be a suitable procedure for patients with negative SLN findings. However, it is unclear whether systematic PLD is needed in patients with positive SLNs. Sentinel node navigation surgery can be performed in these patients. Detected SLNs should be sent to a pathologist for frozen section diagnosis. If lymph node involvement is detected, systematic PLD could be performed [10,13]. However, the European Society of Gynecological Oncology guidelines recommend that PLD and radical hysterectomy should be avoided when lymph node involvement is detected intraoperatively [14].

In this study, PLD with SNB was performed before 2017. After 2017, SNNS was proposed for patients with a tumor size of ≤2 cm. PLD without SNB was performed in patients with a tumor size of >2 cm. Consequently, patients in the PLD group had higher stage and larger tumors according to the change in inclusion criteria. Only one patient in the SNB group had SLN metastasis. Therefore, the benefits of SNB in patients with positive SLN remain uncertain. In contrast, PLD was not required in patients with negative SLN because there were no recurrences in the SNB group. Moreover, the detection rate of SLN was extremely high. We believe that this rate could have reflected the expertise of the surgeons. Furthermore, after 2017, we performed SNB with the removal of the lymphatic vessel for SNNS. This method is safe, accurate, and yields for SLN mapping and dissection [3]. Therefore, SNB may represent an important prognostic factor in the SNB group.

This study has a few limitations that cannot be overlooked. First, the sample size was relatively small for subgroup analysis. Second, there was a bias when assigning patients to either the SNB or PLD group; patients in the PLD group had larger tumors and more advanced disease than those in the SNB group. Therefore, our results should be confirmed in future studies.

## 5. Conclusions

In our study, no disease recurrences or deaths occurred in patients with early-stage cervical cancer with tumors ≤2 cm who had undergone minimally invasive radical hysterectomy with SNB. Therefore, PLD may not be needed for these patients.

## Figures and Tables

**Table 1:** Characteristics of the patients who underwent minimally invasive radical hysterectomy with SNB and PLD.

	SNB	PLD	*p* Value
Number of patients	52	52	
Age *, years old	46 (41–70)	45 (36–51)	0.3
FIGO staging			0.03
1A2	8	3	
1B1	43	43	
2A1	1	6	
Pathological analysis			
Histological type			0.5
Squamous cell carcinoma	24	27	
Adenocarcinoma	28	25	
Tumor size, mm *	12 (7–20)	20 (13–25)	0.001
Large tumor size (>4 cm)	0	1	0.5
Lymph node metastasis	1	9	0.001
Parametrial invasion	0	2	0.5
Deep stromal invasion	7	17	0.02
Lymphovascular space invasion	5	13	0.03
Positive vaginal cut end	0	1	0.5
No adjuvant therapy	45	28	<0.001
Adjuvant RT or CCRT	6	16	
Adjuvant chemotherapy	1	12	
Follow-up, months *	42 (24–60)	82 (19–101)	<0.001
Recurrence	0	4	0.04
3-year DFS	100	91.5	0.04
3-year OS	100	100	0.5

* Median (interquartile range); SNB, sentinel lymph node biopsy; PLD, pelvic lymphadenectomy; FIGO, International Federation of Gynecology and Obstetrics; RT, radiotherapy; CCRT, concurrent chemoradiotherapy; DFS, disease-free survival; OS, overall survival.

**Table 2:** Clinical characteristics of the four patients with recurrent disease.

	Case 1	Case 2	Case 3	Case 4
Age, years old	68	45	46	52
FIGO staging	1B1	1B1	1B1	1B1
Pathological analysis				
Histological type	SCC	AD	SCC	AD
Tumor size, mm	18	14	20	11
Large tumor size (>4 cm)	-	-	-	-
Lymph node metastasis	-	-	-	-
Parametrial invasion	-	-	-	-
Deep stromal invasion	+	-	-	-
Lymphovascular space invasion	+	-	-	-
Positive vaginal cut end	-	-	-	-
Adjuvant therapy	CCRT	-	-	-
DFS, months	9	6	8	10
OS, months	84	49	104	22
Recurrent site	Lung	Lung	Pelvis	Vagina
Outcome	Alive	Died	Alive	Alive

FIGO, the International Federation of Gynecology and Obstetrics; SCC, squamous cell carcinoma; AD, adenocarcinoma; CCRT, concurrent chemoradiotherapy; DFS, disease-free survival; OS, overall survival.

**Table 3:** Subgroup analysis of the patients with a tumor of ≤2cm.

	SNB	PLD	*p* Value
Number of patients	44	30	
Age *, years old	47 (42–55)	45 (35–50)	0.2
FIGO staging			0.2
1A2	8	2	
1B1	35	26	
2A1	1	2	
Pathological analysis			
Histological type			0.7
Squamous cell carcinoma	20	15	
Adenocarcinoma	24	15	
Tumor size, mm *	10 (7–17)	15 (10–18)	0.08
Large tumor size (>4 cm)	0	0	
Lymph node metastasis	0	4	0.01
Parametrial invasion	0	2	0.5
Deep stromal invasion	4	7	0.1
Lymphovascular space invasion	4	6	0.2
Positive vaginal cut end	0	1	0.2
No adjuvant therapy	41	20	0.02
Adjuvant RT or CCRT	2	4	
Adjuvant chemotherapy	1	6	
Follow-up, months *	43 (24–60)	84 (62–105)	<0.001
Recurrence	0	4	0.02
3-year DFS	100	86.4	0.02
3-year OS	100	100	0.4

* Median (interquartile range); SNB, sentinel lymph node biopsy; PLD, pelvic lymphadenectomy; FIGO, International Federation of Gynecology and Obstetrics; RT, radiotherapy; CCRT, concurrent chemoradiotherapy; DFS, disease-free survival; OS, overall survival.

## Data Availability

The data presented in this study are available upon request from the corresponding author.
